# Hypodermic Medication

**Published:** 1871-06

**Authors:** W. Greene

**Affiliations:** Americus, Ga.


ATLANTA

### Medical and Surgical Journal.

NEW SERIES.

Vol. IX. JUNE, 1871. No. 3.

### *Hypodermic Medication.* By W. Greene, M. D., Americus, Ga.

[Read before the Georgia Medical Association at the Meeting in Americus, April, 1871.]

A few years since I wrote an article on "Hypodermic Administration of Medicinal Agents," which was published in the *Atlanta Medical Journal* (May, 1867), and, so far as I know, was the first article on this subject published in any medical periodical of this country, and, strange to say, I have not since seen the subject *practically* treated by any author.

Many of my professional friends then thought I extolled this method of administering medicines extravagantly. I have continued, however, to use the Hypodermic Syringe more extensively, and not only have no reason to change my views therein expressed, but am well satisfied I failed to do the subject justice.

It is a matter of surprise that some of the members of the medical profession entertain a prejudice against the use of subcutaneous medication.

To account for it, I have sometimes thought it results from a lack of a little experience in the use of the Hypodermic Syringe. I am sure that when judiciously used, it is just as safe as medication *per anum*, *per os*, or *per* any other way, and is certainly much more rapid, constant and efficient in its effects. It not only relieves pain CERTAINLY, but it sometimes cures, and cures permanently and radically—nay, *frequently* does so. I am also surprised that even leading medical journals contain so little on this subject. *Ranking's Abstract* and the *American Journal of the Medical Sciences*, for the past three or four years, barely allude to it. The same may be said of the *Practitioner*, *Richmond and Louisville Journal*, *New Orleans Journal*, and all others I have had an opportunity of reading.

It is gratifying to know that my endeavors in this direction have been kindly received by the profession, and their appreciation manifested by requesting my further views, based upon more extensive experience, and guided by the experience and discoveries of others, whose results I have watched and read with great interest.

It is my present purpose again to call attention to this important and interesting subject. I shall endeavor to be practical in what I write, giving no facts that are not sustained by experience or well authenticated, for I am well satisfied that much, perhaps most, that is published in our medical journals of the present day contains too much speculative theory, and that too many medical men write for mere notoriety.

Medical journals should be addressed to the requirements of physicians, whose engagements do not permit them to search over the immense fields from which the fruit has been gathered; to practitioners in the country, as conveying the results of the active labors of the "toilers" in the profession. Of course, I do not assume to lecture editors of medical journals, but I long to see them contain matter more practical, more instructive to the mass of doctors, and thereby more useful and valuable to the country.

The following points in Hypodermy are well established, and I invite particular attention to their consideration:

1st. Clear neutral solutions alone should be used, as they rarely produce irritation.

2d. Whether administered hypodermically, or by the mouth or rectum, the chief physiological and therapeutical effects are the same *in kind*, yet varying *in degree*.

3d. Some drugs, when administered hypodermically or subcutaneously, produce certain symptoms which are absent when administered by other methods, and certain unpleasant syptoms are thus avoided which would interdict the use of perhaps an indispensable treatment.

4th. Rapidity of absorption, and intensity of effect, as a general rule, may always be anticipated.

5th. Locality of injection, whether near or remote from the point affected, as to effect or relief, makes no difference.

Then the advantages to be derived from this mode of introducing drugs are (*a*) rapidity of action; (*b*) intensity of effect; (*c*) facility of introduction; (*d*) with some medicines avoidance of unpleasant sypmtoms. Therefore, when rapid and decided effects of drugs are desired, this mode of administration ought to be adpoted.

The medicines that have been principally employed by the hypodermic method are Aconitine, the preparations of Opium, Atropia, Quinine, Strychnine, Conia, Podophyline, Iodide of Potassa, Bichlorid of Mercury, Prussic Acid, Colocynth, Aloes, Veratrum, Viride, Calabar Bean, Tr. Cannabis Indicæ, Tr. Hyosiamus, and Ergotine. Those in general use, and whose effects are best understood, are Quinine, Morphine, Atropine, Veratrum, Calabar Bean, Ergotine, Aconitine, and the Bichlorid of Mercury, the last experimentally and its practical value not yet determined. I propose to speak of these particularly, giving my observation of them in detail, and the diseases and conditions for which they have been prescribed.

Quinine—While I would not discard it from hypodermic use, must be prescribed with great caution. It often produces violent ulceration, destroying the tissue beneath the skin, and presenting to view the uncovered muscles, conveying to the mind the idea of the action of some corrosive poison. And it has produced this effect in my hands when simply suspended in water, without any of the dissolving acids. I attribute this unpleasant and unfortunate result of the use of Quinine to its *great insolubility*, and hope some means may be discovered by which these unhappy effects may be overcome. Another very serious obstacle to its use is the liability of producing Tetanus, which has, however, never been the case in my own experience, but has occurred in the hands of others, of which I will give a well authenticated instance:

M. Arnold, a military surgeon at Constantia (Algeria), who had used Quinine successfully in ague, warns the profession as to Tetanus being induced by such injections, and mentions two cases—a child and an adult—who both died of Traumatic Tetanus from hypodermic injection of Sulphate of Quinine, dissolved in water and a little Sulphuric Acid. Both patients were suffering from ague. I would, therefore, recommend great caution in the indiscriminate use of this medicine hypodermically.

There are, however, circumstances under which I would not hesitate to use it, and do use it. Where life is endangered by the recurrence of a congestive chill, and my patient will not tolerate Quinine by the stomach or rectum, or can't swallow it, or refuses to do so, or I have not *time* to wait for its constitutional effects by the ordinary method of administration, then, I repeat, I would not hesitate to take the *chances* of ulceration or Tetanus.

The following is the solution of Quinine I employ:

℞—Sulph. Quinine..................gr. xxx.
Sulph. Acid, *dilut*...............gtt. x.
Water (boiling)................℥ ss.—M.

Sig.—Inject from one-half to one drachm half hour before the paroxysm.

The preparations of Opium and Belladonna deserve special mention, because of the universal and almost unlimited use by the hypodermic method for the relief of pain and many of the distressing symptoms of disease, the effects of which are as satisfactory as they are astounding. While diseases differ from each other materially, in cause, symptoms and severity, there is one characteristic in common, which is *pain* (ever present and harrassing), at the bedside by day, unappeased by night, a phantom that walks by the side of the physician, ever ready to bar the door of the sick chamber against his entrance. In these preparations we possess a therapeutic weapon that speedily overcomes and vanquishes this dread enemy.

In my former article, I recommended the use of the Acetate of Morphine, but now use and prefer the Sulphate, becase it is more soluble, and consequently less irritating.

The following is my solution:

℞—Sulph. Morphia................gr. xviii.
Sulph. Atropia................gr. i.
Carbolic Acid.................gr. $\frac{1}{3}$.
Water.........................℥ i.—M.

Sig.—Ten drops equal to one-half grain of Morphine, and one thirty-sixth grain of Atropia.

The Carbolic Acid produces a perfect solution without the further addition of other acids, and prevents the cryptogamic deposit or growth that you find in the ordinary solutions of Morphine without it—thus enabling us to keep a larger quantity of the solution on hand, though it is best to use a fresh solution whenever practicable.

The number of drops calculated for an ounce is three hundred and sixty (360), and not four hundred and eighty (480) as ordinarily supposed; the test of the drop being the amount that passes off as such from the point of the hypodermic needle. There ought to be a more reliable standard of the drop than now exists, and the needles of the syringe should always be the same size. Those manufactured by Lier and Charrier, of Paris, are of precisely the same size; and also, those prepared by Tieman & Co., of New York. I use the *Sulphate* of Atropia, because it is the most soluble and equally as efficacious. As will be perceived, I combine the Atropia in this solution for obvious reasons. The question of antagonism between Opium and Belladonna, and their preparations, has excited considerable controversy during the past few years. I have not time to enter into a full discussion of this question, but will mention that it seems to be the result of extensive observation by learned men of the profession: that Morphine and Atropine are naturally opposing poisons; but that while, on the one hand, Atropine diminishes or removes the hurtful influence of Morphine on the brain, without diminishing its sedative and pain-allaying power, yet, on the other, there is no complete antagonism between the two agents over the whole range of their action. In certain functions of the organism, the antagonism is incomplete—in others it is wholly absent—and in respect of the urinary bladder the effects of the two poisons are alike. It seems, then, that the evidence of antagonism is inconclusive; yet, in my opinion, the physician who does not administer the one, in case of poisoning by the other, FAILS TO DO HIS DUTY. The success depends upon the prompt administration of a dose, or amount adequate to the case in hand; and there can be no other rational guide to this than the quantity of poison taken and probably absorbed, as evinced by existing symptoms. The combination of the Atropine in the Morphine solution seems to be valuable then in many respects. The essential effects of Opium are both intensified and prolonged by the concurrent action of Belladonna. It prevents the nausea frequently, that would otherwise follow the use of Morphine. This combination, as a general diffusable stimulant, surpasses all other drugs, and is a most appropriate and hopeful therapeutic means in all diseases where there is failure of the heart's action, *i. e.* depression of the sympathetic nerve force. It must be borne in mind that an excessive dose will produce depression from over stimulation. Given with a view of exciting or sustaining the heart's action, the dose will range from the 1–100 to the 1–60 of a grain of Atropia, and should never exceed the 1-40. It is especially in cases of the neuralgias—*of pain of every character*—that the administration of this solution exhibits to most advantage. The effects of a few drops of this solution in a case of facial neuralgia or hepatic colic, for instance, injected beneath the skin of the forearm into the areolar tissue, are sometimes almost marvelous—usually within ten miuutes the patient is quite free from pain. The effect of one such dose is startling to any one who has not had some experience in these cases—the beneficial influence continuing for several hours—and, in ordinary cases, not arising from organic disease, effecting a cure. It is remarkable that Morphine thus used has a more *permanent* effect in allaying pain than when given in any other way. After a fracture has been reduced, and the limb placed in a proper position, a small dose injected into the areolar tissue of the limb is of great value in preventing muscular spasm, and, I think, ought never to be neglected. In the distressing vomiting from sea-sickness, which sometimes imperils life, I imagine this solution injected over the region of the stomach would produce charming relief, as it certainly does in other vomitings. I have seen hiccough complicating pneumonia, which had resisted for days various treatment, and strongly threatened death, instantly relieved by injecting six drops of this solution into the intercostal region. Puerperal convulsions after labor are almost always relieved by the injection of ten drops in the arm. In the distressing paroxysms of Asthma, I have never found any remedy so efficacious. In Obstetric practice I find the sub-cutaneous injection of this solution very useful under the following circumstances and conditions, viz:

1st. During painful dilutation and expulsory periods, especially in primiparæ and in narrow pelves. 2d. In spasmodic pains· 3d. In painful complications of the process of labor in general. 4th. In severe after pains.

It is in the first category of cases that I have especially employed it, injecting one or both thighs. I might go on and occupy pages, enumerating the conditions and circumstances where these drugs have been employed with complete satisfaction.

I will only further state that wherever *pain* is present, which no human being ever willingly endured for a single moment, always use the hypodermic syringe. In children and delicate females I employ the compound liquor of Opium, prepared by Dr. Edward R. Squibb, in doses from five to fifteen drops, or more—always beginning with a minimum and gradually going to a maximum dose. There is no risk whatever in using it on children—even infants. In small children, I usually introduce the medicine under the skin at the back of the neck.

The pain inflicted is of no consequence, and there is much less annoyance than pouring it down their throats. The tolerance of the human system to the preparations of Opium, administered hypodermically, is very surprising, and I shall briefly allude to it. There are several cases on record, well authenticated, of employing it to such an extent that the body was perfectly covered by the points of puncture.

There is a young man in this city, who, during the past three years, has had the instrument used upon himself about *four thousand* times. He had Scarlitina when a child, and the inflammation extending from the tonsils and fauces to the eustachian tube, resulted in disease and ulceration of the bones of the ear, causing most violent paroxysms of pain. He could not take any of the preparations of Opium by the stomach without the greatest distress, and nothing else seemed to give him any relief. The Hypodermic Syringe was called into requisition, and Squibb's Compound Liquor of Opium, in twenty drop doses, first used with immediate and marked relief whenever the attacks come on. He visited and consulted the most eminent medical men in the United States, and permanent relief was pronounced by them impossible. After various medication, it was decided to direct remedies alone to the relief of these paroxysms of pain—not supposing the unfortunate young man could much longer survive. Thus he has gone on, using the hypodermic injections daily, generally five or six times in twenty-four hours for about three years, occasionally increasing the dose, until now it only requires about one grain of Morphine and 1-30th grain of Atropine at each injection.

He does not seem any worse now than two years since, except some loss of flesh, and a weakening of his intellectual sensibilities, together with an impairment of vision, requiring the use of glasses in reading, produced by the Atropine. He carries about his person constantly, wherever he goes, a hypodermic syringe and solution of Morphine and Atropine, to be used whenever an attack comes on; and, so common is it in the city, that any one who first gets to him uses the instrument—even a negro servant who often accompanies him. He carries a little blank book in his pocket, properly ruled, where the time of day and number of drops of the solution are recorded and referred to each time before giving him another injection, as a guide to the amount necessary to be used at that time.

This is one of the most remarkable cases on record of the use of hypodermic injection. How much longer he will survive in this unfortunate condition, no one can tell. He is daily about town, and can walk several miles. It is remarkable that he has not suffered more local trouble from these frequent punctures. They have been made almost exclusively in his arms from his shoulders to his hands, over and over again, until the areolar tissue is very much thickened and indurated, and has only had an occasional abscess which gave no further trouble after the pus had been discharged.

This case convinces me of the importance and value of the combination of Atropine with Morphine when used hypodermically.

I find that it does not require such constant and rapid increase of the Morphine, and there is less risk of a patient becoming addicted to its habitual use, when thus combined. In view, then, of these facts, and the unlimited calls that may be and are made upon the physician, where the hypodermic use of remedies can be so advantageously employed, is it not highly important that he should always have a standard solution at hand (the strength and effects of which he is well acquainted with) that he may quickly and safely apply it in cases of emergency?

I would suggest that every physician have a hypodermic case, which he can either carry in his pocket or medicine chest, containing a syringe with two or three needles and four vials (drachm) filled with the solution of Morphine and Atropine, Squibb's Compound Liquor of Opium, Norwood's Veratrum Viride, and Oxalate of Cerium—the last to relieve nausea when produced by any of the other medicines, given in doses of four or five grains on the tongue. I find nothing better. I would not feel that I had performed my duty if, in this connection, I failed to caution my professional brethren concerning the indiscriminate use even of these drugs. Atropia is a powerful poison, the physiological action of which has of late years been the subject of careful experiment and research; the most recent and able writers being Dr. John Harley and Dr. Meuriot. In poisonous doses, the frequency of the respiratory movements are *decreased*, though paralysis of the pneumogastric nerves, producing restlessness, insomnia and delirium, until the patient passes into *coma* and dies. The best antidotes are Morphia and Extract or Tincture of Calabar Bean—and, I repeat, that the physician who does not use the former *fails to do his duty*, notwithstanding the conflict of opinion on this subject among our learned professors. I will directly speak more particularly of the Calabar Bean. Morphia, though rarely deceiving our most sanguine expectations, is not altogether harmless, and may produce frightful symptoms. I will mention a case:

Mrs. H. C., aged thirty-five, in good health otherwise, had been kept awake for seventy-two hours by intense neuralgic pain on the left side of head, face and neck, arising from a carious molar tooth on left side of lower jaw. She was injected at one A. M., June 28, with one-third of a grain of Acetate of Morphia, dissolved in about four drops of water under the skin of the left arm, just over the insertion of the deltoid muscle. No blood appeared at the puncture. In about fifteen seconds tightness of the chest and difficulty in breathing were complained of, and the patient asked to be raised, saying she felt as if she were dying.

Her face and lips now became pale; speech became indistinct (not inaudible); pulse irregular; some spasms of the facial muscles took place, and she fell back to all appearances dead. Cold water was freely dashed over her face and chest, and, as she was unable to swallow, her tongue was rubbed over with Sal Volatile, and Ammonia applied to her nose, artificial respiration being kept up all the time. During the while her face was bleached, pulse not to be felt, and respiration not to be perceived. Insensibility continued for about three minutes, then, happily, one or two feeble beats of the pulse, and a shallow inspiration or two, showed returning animation. She then became conscious, pulse feeble and irregular; respiration slow; fingers remained numbed, and both thumbs were firmly drawn into the palms of the two hands. This passed off in about six minutes, leaving her feeling very ill, but free from the neuralgic pain which did not return.

There was no feeling of nausea, and no attempt at vomiting during any part of the time.

I will now leave the discussion of this interesting part of the subject to speak briefly of the physiological and therapeutical effects of the Calabar Bean employed hypodermically, which presents many interesting and curious developments, and opens an extensive field for research and experimental investigation.

During the last few years M. Bournville, house surgeon to the Paris hospital, has employed it with care and studied its effects on several diseases. Among the results may be mentioned the following: Every one knows that the Calabar Bean, when instilled into the eye, produces a very remarkable contraction of the pupils. Now he states that it is surprising to observe that, when it is injected under the skin, there ensues, on the contrary, a dilatation of the pupil, or else the ocular diaphragm remains unchanged. A second point which he notices is the antagonism between the Calabar Bean and Atropine. He verified this phenomenon in six guinea pigs. After having injected the Bean, Atropine was injected. The animals did not succumb when both medicaments were injected in fit proportions; but two or three days afterwards, a dose of the bean equal to that which had been at first employed, being injected into the surviving animals, *they all died.*

This obviously shows the antagonistic action of Atropine against the Calabar Bean. He also confirmed these experiments on a human subject, and treated with more or less success Epilepsy, Chorea and Tetanus.

Dr. Eben Watson states that in all his experiments the pupils remained either unchanged or were dilated.

Mr. T. Wharton Jones, in a suggestive paper, published in the *Practitioner*, for September, 1869, refers to the surprising fact and says: "The Calabar Bean is the direct antagonist of Atropine," and asserts that "the local application of the drug causes contraction of the veins, and Atropia of the arteries."

Dr. Alois Monti, of the St. Anne's Child's Hospital, reports three cases out of five of Trismus Neonatorum cured by this remedy. He prefers the hypodermic method, because he considers the internal use uncertain. He repeats these injections every ten or fifteen minutes until the spasms cease; and then intermits them even for several hours until the cramp returns again. For new born children, he uses one-tenth grain of the extract for a dose, and goes up to one-third, one-half or a whole grain a day. Older children can commence with one-third of a grain for a dose. For internal use, one to four grains a day may be given.

In the *Richmond and Louisville Journal*, for August, 1870, Dr. F. C. Wilson reports a case of Epilepsy in which there had been six successive convulsions at intervals of twenty minutes, relieved by ten minims of the Tincture of Calabar Bean used subcutaneously.

In the *Medical Examiner*, for July, 1870, Dr. Newman reports a case of Tetanus successfully treated by the hypodermic injection of Calabar Bean.

The *Glasgow Medical Journal* (1869, p. 300), publishes two cases treated by Dr. William Fenwick. The first case died in less than thirty-six hours after the commencement of treatment, owing to the culpable negligence of the attendants; the directions being very ineffectually carried out. The second case recovered, and is especially interesting, owing to the suspension of the medicine being three times followed by increase of symptoms, which yielded to its renewed exhibition.

Dr. Eben Watson, in the *Practitioner* of September, 1870, in a paper "on treatment of Tetanus by the Calabar Bean," details several cases—eighteen, I believe, in which he reports ten recoveries—and always found the remedy to act decidedly better when introduced under the skin; in fact, considers this the most essential point in its administration. Thus, we perceive a wide and interesting field for the hypodermic use of this medicine. My own opportunities have been limited for its exhibition. In a case of Epilepsy, I found it exceedingly efficacious. I have drawn from reliable and trustworthy authority, and hope sufficient evidence has been adduced to induce my professional brethren to test its virtue and efficacy.

I will now proceed to speak of Veratrum Viride, a most familiar remedy, and am sure there is no medicine in our Materia Medica that deserves more praise, and whose effects on the human system are more uniform and consequently more to be relied upon. All are acquainted with its effects upon the human system when administered by the mouth, and it is, therefore, to its hypodermic use that I invite your attention. It effectually takes the place of the lancet when thus administered, and acts more rapidly, more energetically, and more satisfactorily. When arterial excitement or tumultuous action of the heart is sought to be controlled, this medicine injected under the skin in proper doses accomplishes a desideratum most devoutly to be wished for. In the convulsions of children, the result of high arterial excitement, so trying to the physician and frightful to the relatives and friends, we possess a certain remedy in Veratrum. I begin the injection under the skin at the back of the neck in half drop doses which may be increased to two drops, combined with a few drops of warm water; and, if its constitutional effects are not produced in ten or twenty minutes, cautiously repeat the dose. I frequently combine with the Veratrum five or ten drops of Squibb's Compound Liquor of Opium. In the first stage of Pneumonia, where it is important to produce rapid sedation, or where the stomach fails to tolerate it, I find it invaluable. I use it cautiously, beginning with one or two drops. I will here relate a case in point, occurring a few days since in the practice of my friend, Dr. Geo. F. Cooper, of this city, in his own words:

"Lucy, mulatto girl, aged sixteen, attacked with double Pneumonia; saw her fourth day; pulse 125; respiration labored and rapid. Injected one drop of Norwood's Veratrum under the skin of arm. In twenty minutes pulse reduced to 118. Injected two drops more. In twenty minutes pulse reduced to 90; then ordered it continued by mouth every three hours, in four or six drop doses. Her recovery, though not rapid, was uninterrupted and complete in eight days. No blister or other medication used, except two or three purgative doses of Mercury."

In Puerperal convulsions, Veratrum is a most valuable remedy. I will give two recent cases in which I employed it, thereby better conveying to the mind the mode of its administration and its effects on the system. January 8, 1871, called to see Mrs. W., aged thirty-eight, in consultation with my friend Dr. Bird, two miles from town. Arrived at her bedside eleven o'clock P. M. She lacked two months to the completion of her term. Nothing unusual had occurred during gestation, her general health being uninterrupted; had borne several children and never required the presence of a physician during her labors. She had been having convulsions since early in the morning, at intervals of twenty to forty minutes; had taken large doses of Laudanum, and been kept under Chloroform all day; bowels been freely opened with Enemata; pulse very large, and as hard a one as I ever felt; it beat 130 times a minute. The *os uteri* was just large enough to admit the point of my index finger, and very rigid.

Had a dreadful convulsion a few moments after my arrival. I immediately opened a large median vein in her right arm and allowed blood to flow until her pulse had sunk very low. She had no return of the convulsions for about an hour, remaining nearly comfortable. When her circulation began to increase, restlessness came on and she had a most violent eclampsia followed by apoplectic stertor, from which she partially recovered in half an hour. I immediately gave her ten drops of the solution of Morphine and Atropine, which had the effect to quiet her at once, and she rested and slept comfortably for one hour and forty minutes. She awoke much excited and another convulsion came on. I determined then to give her Veratrum subcutaneously. By this time she refused to open her mouth or swallow, the stupor being more profound than before. This was fifteen minutes past two o'clock; pulse 140; injected two drops of Norwood's Veratrum. In ten minutes pulse reduced to 130, with no disposition to go lower; half-past two o'clock injected three drops more. In fifteen minutes slight impression noted on pulse; convulsions strongly threatened. Fifteen minutes of three o'clock injected four drops. Three A. M. pulse reduced to 90 beats; breathing much improved, and sleeping quietly. Awoke at six A. M. perfectly rational, with ability to swallow. Directed following prescription:

℞—Bromide Potass....................℥i

Water..........................℥viii—M.

Sig.—One table-spoonful every six or eight hours, in half glass water.

The circulation was kept under control for two or three days by occasional doses of Veratrum, combined with the Bromide. She continued to do well until February 12th, when I was sent for and she was delivered of a dead fœtus of eight months. Labor was rapid and easy, from which she recovered as usual. Now could anything be more charming than the prompt result of this medicine, and that after the much vaunted remedy (bleeding) seemed to fail? The Veratrum did not produce the slightest nausea. Dr. Bird rendered valuable assistance in conducting this case.

[Since this article was commenced, I have witnesied a most terrible case of Puerperal convulsions, the treatment of which presents many interesting features. I will merely give my notes of the case as taken down at the bedside:]

Called, March 5th, 11 A. M., to see Mrs. H. in consultation with her attending physician, Dr. Hudson. She was a woman of a very susceptible, nervous constitution, and labored under constitutional irritation, from the commencement of her pregnancy. She was 25 years old, and this her first labor—had been married about twelve months; was expecting her labor every hour, the time being out; had complained of intense headache for several days. About 4 A. M., she awoke her husband, to do something for her head. By the time he could make a light, she had a convulsion; had no uterine pain whatever. The convulsions returned every ten or fifteen minutes, until Dr. H. arrived (about an hour afterwards), and gave a large dose of Morphine, and put her under Chloroform, when the convulsions were delayed an hour or more in their intervals. About 10½ o'clock they became more rapid, and I was sent for. She had a convulsion when I arrived, and immediately sunk into a stertorous, apoplectic sleep. I felt that the fatal blow had been struck, effusion or extravasation having taken place. There was no uterine action, the os uteri was firmly contracted, very rigid, and the perineum excessively hard and thick; the vertex in the first position. The fœtal heart could be distinctly heard; withdrew the Chloroform, bled her eighteen ounces, and gave ten drops of solution of Morphine and Atropia, fifteen minutes thereafter; had five convulsions in quick succession, the last one lightest. Eleven and a half, A. M., pulse 145; injected one drop Veratrum in a few drops water; in ten minutes pulse reduced to 130; in twenty minutes, 120; at twelve and three-quarters, convulsions. Eight minutes to 1 o'clock, P. M., gave one drop Veratrum combined with eight drops solution of Morphine and Atropia, pulse 115; 1.25 P. M., pulse 135, and convulsion came on; bled her twelve ounces; 1.32, P. M., injected one-half grain Ergotine combined with two drops Veratrum, in thigh, pulse being 135. In eighteen minutes, pulse reduced to 120; twenty minutes, no change; twenty minutes more, pulse 118; two and a half o'clock, convulsion; seven minutes after three, pulse 128, os uteri yet contracted and no uterine pain, conea being profound and stertorous, rapid breathing—child alive. Three, P. M., ruptured membranes, and injected one-half grain Ergotine in thigh, combined with three drops Veratrum. Forty-five minutes after three, pulse 120; injected three drops Veratrum, in ten minutes pulse 116, and gave one-half grain Ergotine. Some uterine contractions—waters all passed off. Os dilated, size, half dollar.

Five P. M., severe convulsion. Forty minutes after five, one-half grain Ergotine injected in abdomen; uterine contractions decidedly increased, and coma *very* profound. Forty-five minutes after six, P. M., convulsion—uterine contraction going on. Seven, P. M., head of fœtus descended so that forceps could be applied, which was immediately done, and a twelve pound child extracted. The mother died in one hour, and child doing well.

This woman never spoke or exhibited the smallest sign of reason or sensation, from the moment of invasion, but sunk at once into the stertorous, apoplectic sleep that finally led to the sleep of death. Notwithstanding, these functions of the brain were oppressed from the beginning, the hemispheres, the cerebellum and the tubercula quadrigemina remaining extinct, as to power; yet, our remedies did much good. This is very interesting. The Veratrum controlled the circulation and lengthened the intervals of convulsions from ten or fifteen minutes to one hour and twenty minutes. The Ergotine speedily and promptly excited uterine contractions, and thus enabled us to save the child. We could not have done so without it; the mother's case already a hopeless one—the clild alive, as the strokes of its heart indicated, profound coma, and total inability to swallow, and no effort of the womb whatever to expel its contents, it did seem that the mother would succumb with a live child in her belly. The Ergotine injected under the skin of the thigh and abdomen aroused this dormant uterus, and it responded by sending the fœtal head down into the vagina, where it could be reached by forceps and delivered.

I am indebted to Dr. Hudson for valuable assistance in this case, and courtesy in obtaining its history.

I have found the addition of Veratrum with the Morphine and Atropine solution, speedily relieve distressing paroxysms of Asthma that had resisted all other treatment.

I have never seen any reference to the hypodermic use of Veratrum, and hope by this imperfect account of its employment to attract the attention of the profession, that its value and efficacy may be further developed and illustrated.

I come now to speak of Ergotine. In controlling excessive menorrhagia there is no remedy equal to it, used hypodermically. Severe cases of this character that had resisted various treatments and strongly threatened death, have yielded almost instantly to the hypodermic injection of Ergotine dissolved in equal parts of water and Glycerine. In my hands it has produced remarkable effect in the hemorrhage following delivery. Under these circumstances of sudden and alarming bleeding we want a remedy that speedily affects the system. I present it to the profession and ask for it a faithful trial. The dose is from one-third, one-half or even one grain dissolved in equal parts of glycerine and water, and the injections repeated every fifteen or twenty minutes. The hypodermic use of Ergotine in aneurisms is attracting considerable attention. I have had no opportunity of testing its value in these cases, but will mention two cases reported by Langenbeck. The first was that of a man aged forty-five, with a pulsating tumor in the neck, the size of a fist, the pulsations in which greatly annoyed the patient. On the 6th of January, 1869, Professor L. made a first subcutaneous injection of 0.03 gr. (half a grain) watery extract of Ergot on a level with the aneurism. After this the pulsations became weaker, and the tumor diminished in size. Between the 6th January and the 17th February, these were injected at regular intervals of about three days, two grammes of Ergotine in doses varying from 0.03 to 0.18 grammes (half a grain to three grains). The tumor was notably diminished in size and the pulsations were less strong.

The second case was that of a man aged forty-two, who had for twenty years a pulsating tumor of the radial artery of the size of a hazel nut, three centimetres from the hand. Professor L. injected 0.15 gramme of Ergotine between the skin and the tumor; the next day the aneurism could not be detected. It reappeared momentarily in consequence of a slight effort made by the patient, but it had completely disappeared at the end of eight days. The editors of the *Archives Generales* (November, 1869), from which journal this account was condensed (see American Journal, 1870,) justly remarks that "these results seem extraordinary and to have been obtained very promptly." I hope to hear of further experiment with this remedy in this disease.

The hypodermic injection of the salts of Mercury, in the treatment of constitutional syphilis is engaging the attention of the profession, espcially in the East. I've had no patients recently who would submit to its use, and, therefore, have no personal experience with it. I am, however, well satisfied of its utility and efficacy, and shall not hesitate to employ it. I will briefly glance at what eminent medical men say of it by way of attracting the attention of the profession to its employment, for a few experiments conducted by master hands in all that relates to therapeutics and pharmacy are more important and reliable than a far greater number would be detailed by persons of less authority.

M. Diday, at a meeting of the Lyon's Medical Society, detailed the result of twelve cases of syphilis treated by hypodermic injection of corrosive sublimate. His solution consisted of distilled water 45 grammes, corrosive sublimate, glycerine, of each ten centigrammes. Two or three injections were practiced daily, the mean quantity of the sublimate introduced being seven or eight millegrammes. The back is used for the operation, because pain is less complained of there—or if the patient injects himself, the sides of the chest and anterior or external parts of the thighs are used, alternating these regions so that the punctures do not succeed each other too rapidly or approach each other too closely. He says: "I must admit great embarrassment in pronouncing any positive opinion on this new method. In fact, there is no occasion to pronounce any positive opinion on a remedy which has so recently made its appearance. At first, one is struck and seduced by the promptitude of some of the cures which it effects." M. Diday believes it most advantageous in the squamous forms of syphilis, and warns against its use in the ulcerative forms. In none of his cases was the slightest mercurial affection of the mouth produced.

Dr. T. J. Walker, *British Medical Journal*, records the results of his experience, in ten cases in which it was fairly tried, and found immediate improvement after one or two injections.

He says: "In the first case, after three weeks of treatment and the injection of only 7–10 of a grain of Bichlorid of Mercury, the change in the condition of the patient was surprising."

In case second these sypmtoms, which were rather of the secondary type, were all relieved after six injections, and the tertiary symptoms yielded with unusual rapidity to the Iodide of Potassa when it was commenced." The solution he employed amounted to one-third grain of corrosive sublimate dissolved in ten drops of water and glycerine. This solution may be used every day. Dr. Walker states that the pain and inflammation at the seat of puncture are comparatively slight, and considers the results he has obtained justify its trial in large institutions.

The Hypodermic Syringe is an exceedingly useful little instrument for drawing out liquors for diagnostic and other purposes. It has been made use of for obtaining blood for examination from cholera patients. It has been used for the operation of transfusion, and with slight modification, preferred above all other instruments. Dr. Mader, of Vienna, used it in making a diagnosis in a case of ascites, in which a doubt existed whether chronic peritonitis or cirrhosis hepatis caused the dropsy. The character of the liquid proved it to be a case of cirrhosis. He also pumped out a chronic serous exudation from the pericardial sac of an aged female. The first operation yielded a few ounces of liquid and relieved the urgent symptoms; at a second trial three ounces were removed. Mader thinks the operation deserves much regard, as it is easily performed, and attended with comparatively little danger. In these operations the canula should be provided with a stop-cock, so that air may not enter when the body of the syringe is detached, in order to empty it, and the body of the syringe should be made of greater capacity.

I will not lengthen this already too extended review, by referring to other remedies that are used hypodermically, and mentioned in the beginning of this article. The field is wide open, and the opportunity for research and investigation inviting, and offert a rich harvest to the industrious lover of science. I feel that I could not practice medicine without the Hypodermic Syringe—know that I am indebted to it for many a night's sleep and relief from restless anxiety. Instead of worrying all night, as I have many times done, with cases of colic, delirium tremens, gout, rheumatism, convulsions, neuralgia, nausea and nervous excitability, I have relieved my patients in fifteen or twenty minutes, and returned to my bed.

