# Erratum

**Published:** 1871-06

**Authors:** 


### *Erratum.*

In the very able paper on "Hypodermic Medication," in the original department of this number of the Journal, the author's name is not correctly given. It should be Wm. A. Green, and not W. Green. We are induced to make this correction as we learn there are more than one Dr. W. Green in the State.

